# Animal Models of Inflammation for Screening of Anti-inflammatory Drugs: Implications for the Discovery and Development of Phytopharmaceuticals

**DOI:** 10.3390/ijms20184367

**Published:** 2019-09-05

**Authors:** Kalpesh R. Patil, Umesh B. Mahajan, Banappa S. Unger, Sameer N. Goyal, Sateesh Belemkar, Sanjay J. Surana, Shreesh Ojha, Chandragouda R. Patil

**Affiliations:** 1 Department of Pharmacology, R. C. Patel Institute of Pharmaceutical Education and Research, Shirpur 425405, Dist- Dhule, Maharashtra, India; 2 Pharmacology & Toxicology Division, ICMR-National Institute of Traditional Medicine, Nehru Nagar, Belagavi 590010, Karnataka, India; 3 SVKM’s Institute of Pharmacy, Dhule 424001, Maharashtra, India; 4 School of Pharmacy and Technology Management, SVKM’s NMIMS, MPTP, Shirpur 425405, Dist- Dhule, Maharashtra, India; 5 Department of Pharmacology and Therapeutics, College of Medicine and Health Sciences, United Arab Emirates University, Al-Ain, PO Box 17666, United Arab Emirates

**Keywords:** anti-inflammatory, phytoconstituents, biomarkers, drug discovery, animal models

## Abstract

Inflammation is one of the common events in the majority of acute as well as chronic debilitating diseases and represent a chief cause of morbidity in today’s era of modern lifestyle. If unchecked, inflammation leads to development of rheumatoid arthritis, diabetes, cancer, Alzheimer’s disease, and atherosclerosis along with pulmonary, autoimmune and cardiovascular diseases. Inflammation involves a complex network of many mediators, a variety of cells, and execution of multiple pathways. Current therapy for inflammatory diseases is limited to the steroidal and non-steroidal anti-inflammatory agents. The chronic use of these drugs is reported to cause severe adverse effects like gastrointestinal, cardiovascular, and renal abnormalities. There is a massive need to explore new anti-inflammatory agents with selective action and lesser toxicity. Plants and isolated phytoconstituents are promising and interesting sources of new anti-inflammatories. However, drug development from natural sources has been linked with hurdles like the complex nature of extracts, difficulties in isolation of pure phytoconstituents, and the yield of isolated compounds in minute quantities that is insufficient for subsequent lead development. Although various in-vivo and in-vitro models for anti-inflammatory drug development are available, judicious selection of appropriate animal models is a vital step in the early phase of drug development. Systematic evaluation of phytoconstituents can facilitate the identification and development of potential anti-inflammatory leads from natural sources. The present review describes various techniques of anti-inflammatory drug screening with its advantages and limitations, elaboration on biological targets of phytoconstituents in inflammation and biomarkers for the prediction of adverse effects of anti-inflammatory drugs. The systematic approach proposed through present article for anti-inflammatory drug screening can rationalize the identification of novel phytoconstituents at the initial stage of drug screening programs.

## 1. Introduction

Inflammatory diseases are globally identified as the major cause of morbidity across the population [1]. Inflammatory condition is associated with the activated immune system, including activated immune cells and the bio-molecules [2]. Inflammation is a defensive response of an organism against invasion by the foreign bodies like bacteria, parasites, and viruses. An acute inflammatory response is manifested as redness, heat, swelling, pain, and the loss of function. Increased vascular permeability, accelerated blood flow, and nerve fiber sensitization are associated with swelling, redness, and pain respectively [3]. The protective effects of inflammatory cascade and potential for tissue destruction are usually balanced in normal state. Whereas, chronic inflammation is usually characterized by substantial destruction and recovery of injured tissues from an inflammatory response [4]. If uncontrolled, inflammation may arise numerous diseased states like rheumatoid arthritis, multiple sclerosis, inflammatory bowel disease, psoriasis, immune-inflammatory ailments, and neoplastic transformations [5,6,7]. Furthermore, chronic inflammation is also linked with various steps of tumorigenesis and recognized as risk factor for the occurrence of different types of cancers [8]. Many chronic diseases manifest due to presence of low grade sustained inflammation. Treatment of the chronic inflammatory diseases like rheumatoid arthritis and inflammatory bowel diseases is still a challenge due to lack of safe and effective drugs [2].

Finding a safe and effective drug to control inflammation has been a challenge and therefore, many animal models have been developed for the evaluation of drugs having anti-inflammatory properties. In the past few years, plant-derived molecules known as phytochemicals or phytoconstituents or natural products appear to be a major source of drugs and being evaluated as a drug candidate for anti-inflammatory actions. Evaluating phytoconstituents in the in-vivo animal models has been a proof of the concept and mainstay of drug development program for the disease specific anti-inflammatory property. Choosing the right animal model for the preclinical evaluation has been a challenge in establishing the efficacy and mechanism of action of drugs for translation as a therapeutic effect in humans. Although numerous in-vivo and in-vitro models for anti-inflammatory drug development are available, the judicious selection of the animal model is always critical and brings a challenge for the inclusion in drug discovery and development. An inappropriate selection of animal models can lead to false positive or false negative results and may limit the discovery of promising drug molecules from a screening program. Therefore, adoption of a systematic approach for the anti-inflammatory screening of phytoconstituents or natural products is highly desirable. Our review comprehensively discuss the different models and mechanisms of inflammation therein and phytoconstituents screened in those models may provide a ready reference for such need. This review include an outline of the inflammatory process, the animal models used to study the etiopathogenesis of inflammation in general along with their merits and demerits for the pharmacological evaluation of an anti-inflammatory drug discovery precisely from natural origin.

## 2. The Need for Newer Anti-Inflammatories

Inflammatory diseases, including different types of rheumatic diseases, are a major healthcare problem worldwide. The majority of human population is affected by inflammation-related disorders. Though several agents are accessible to treat multiple inflammatory diseases, their prolonged use leads to serious adverse effects [9]. Management of inflammatory diseases either with steroidal or non-steroidal drugs is a traditional clinical practice. The non-steroidal anti-inflammatory drugs (NSAIDs) inhibit early steps in the biosynthesis of prostaglandins through the inhibition of cyclooxygenase (COX). The NSAIDs are important drugs used to reduce untoward consequences of inflammation [10]. The chronic use of NSAIDs is connected with cardiovascular, gastrointestinal, and renal toxicities [1,11,12,13,14]. Similarly, the use of corticosteroids leads to hypertension, hyperglycemia, osteoporosis, and growth arrest [15]. Toxicity and recurrence of symptoms on discontinuation is a major problem related to currently available synthetic drugs [10]. The development of safer anti-inflammatory agents remains to be a subject of great interest [16].

Development of anti-inflammatory drugs derived from natural sources is the rational and productive strategy towards the cure of inflammatory ailments [11]. Natural products are safe, efficacious, biocompatible, and cost-effective alternatives to treat inflammatory diseases [17]. Many countries like India, China, Brazil, and Sri Lanka have a rich heritage of using natural products in therapeutics as the traditional medicine since ancient time and also provide a time tested safety of these plant-based medicines. Currently the experimental studies are demonstrating the molecular and pharmacological mechanisms accountable for their health benefits and therapeutic prospects. Integration of traditional knowledge and indigenous resources will assist the development of novel anti-inflammatory leads [1]. Many scientific studies on plant species which has been used as folk medicine against the inflammation have established the recognition of natural products as potential anti-inflammatory drugs [18]. The anti-inflammatory effects of phytoconstituents are exerted through their action on key regulatory molecules, including cyclooxygenase (COX), inducible nitric oxide synthase (iNOS), and cytokines [14,19].

## 3. The Inflammatory Cascade

**Figure 1:**
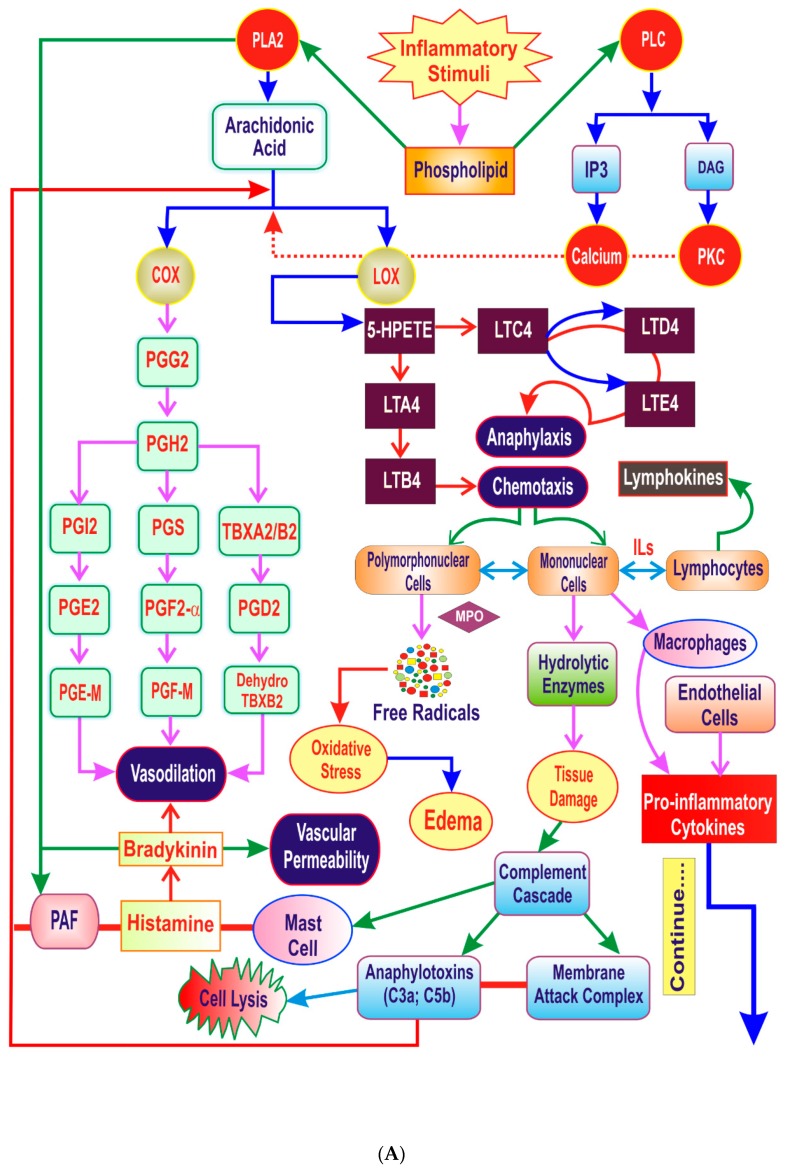

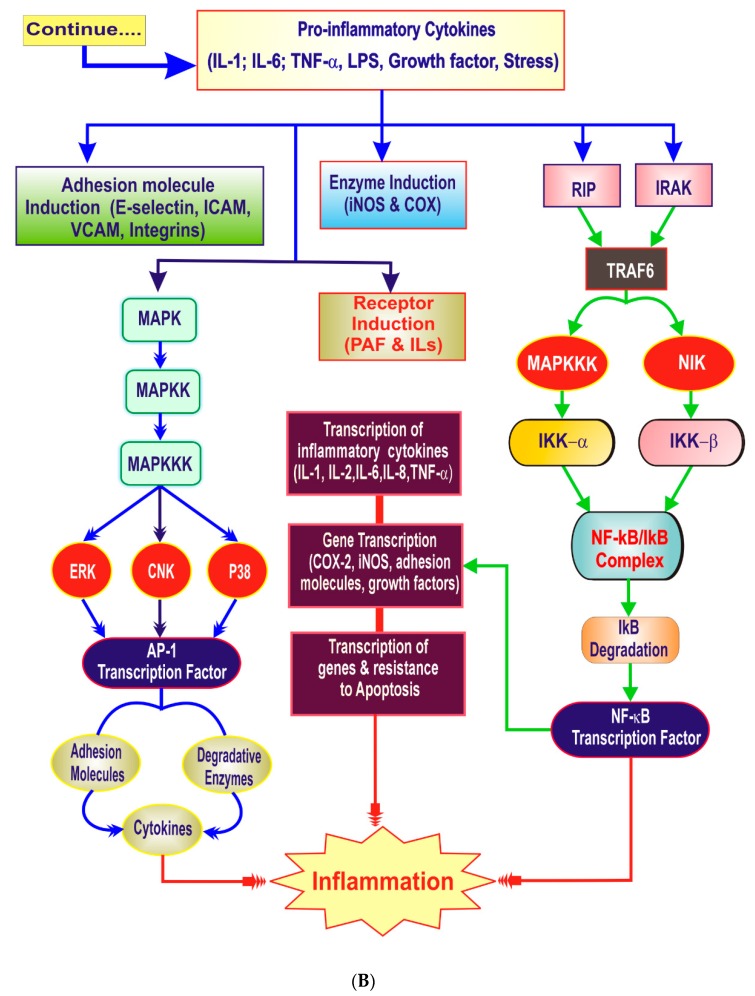
(**A**) The inflammatory cascade; (**B**) The inflammatory cascade. The arrows in the figure represents process. The arrows in the figure represents process

Inflammation involves localized rise in leukocyte number and many complex mediators. Prostaglandins are universal substances which indicate and modulate the tissue responses during inflammation. Biosynthesis of prostaglandins has been implicated in the pathology of Alzheimer’s disease, cardiovascular diseases, colonic adenomas, and cancer [20,21]. The inflammation is either acute or chronic and occurs in three distinct phases. Inflammation starts from an increased vascular permeability, infiltration of leukocytes, followed by granuloma formation and tissue repair [22]. Arachidonic acid metabolites, adhesion molecules, cytokines, chemokines, and platelet-activating factor cause release of other mediators and initiates chemotaxis [3]. The microbial products and host proteins like complement proteins, kinins, and coagulation system activates the production of inflammatory mediators. Various inflammatory mediators like complement proteins and kinins originate from plasma, whereas histamine, prostaglandins and cytokines originate from cells. Arachidonic acid metabolites like leukotrienes (LTs), prostaglandins (PGs), and 12-Hydroxyeicosatetraenoic acid (12-HETE) are actively involved in the development of inflammatory diseases, like asthma, arthritis, and cancer [23,24]. Inflammation is a result of activated cellular elements and the existence of various biochemical mediators like cytokines (e.g., Interleukin-1, TNF-α), Kinases (p38 kinase, JNKs, MAP kinase), transcription factors (e.g., NF-κB) and matrix metalloproteinases (MMPs) [2]. The inflammatory cascade is shown in Figure 1A,B.

## 4. Key Players of Inflammation

### 4.1. Lipid Derived Mediators

Arachidonic acid (AA) is a chief eicosanoid precursor and a basic constituent in all the cells of body. Activation of various phospholipase enzymes, mainly phospholipase A2 (PLA2), causes release of AA from membrane phospholipid. AA is metabolized via different pathways and form multiple oxygenated products called as eicosanoids. The cyclooxygenase (COX) forms prostaglandins (PGs) and thromboxane (prostanoids) whereas the lipoxygenases (LOX) forms leukotrienes (LTs) and lipoxins (LXs). Additionally, cytochrome P450 enzymes produces epoxyeicosatrienoic acids (EETs) [25]. Eicosanoids regulate various inflammatory and homeostatic process that is linked to many diseases [26]. The proinflammatory activity of lipid-derived mediators has been well documented [27]. The PGs are connected with bronchoconstriction, mucus secretion, vasodilatation, and vascular permeability. Whereas, leukotrienes are putative bronchoconstrictors and stimulators of vasodilatation and vascular permeability [28]. Leukotriene B4 (LTB4) is associated with neutrophil activation and superoxide formation. Furthermore, it increases interleukin-6 (IL-6) production and stimulates the early gene transcription of other cytokines [28,29]. The 5-lipoxygenase (5-LOX) is vital for the leukotrienes biosynthesis and LTs are key mediators in an inflammatory and the allergic processes. Therefore, inhibition of 5-LOX is promising approach for the treatment of dermatitis and psoriasis [30]. Platelet-activating factor (PAF), generated by various inflammatory cells including macrophages, neutrophils, eosinophils, and endothelial cells is another mediator that causes bronchoconstriction, platelet activation and chemotaxis [28].

### 4.2. Proinflammatory Cytokines

Cytokines regulate the immune responses and inflammatory process. Tumor necrosis factors, interferons, interleukins, and colony stimulatory factors belong to the class of cytokines. Cytokines regulate adhesion molecule expression, cell growth, cell division, apoptosis, immunoglobulin production, and chemotaxis in the target cells [28]. Stimulation of monocytes and macrophages releases proinflammatory cytokines like tumor necrosis factor- α (TNF-α), IL-1β, and IL-6. TNF-α is involved in the tumor cell metastasis and pathology of rheumatoid arthritis [31]. IL-1β activates lymphocytes and causes bone resorption [2]. The TNF-α and IL-1β regulate the expression of adhesion molecules and also captures the circulating leukocytes [32]. Furthermore, cytokines also initiates intracellular signaling cascades and subsequent transcription [33].

### 4.3. Vasoactive Mediators

Histamine, stored primarily in the mast cells and basophil leukocytes is a widely distributed and preformed proinflammatory mediator [28,34]. Release of histamine causes a transient increase in permeability following tissue injury. Histamine causes endothelial cell contraction and allows the passage of fluid along with the proteins through the inter-endothelial junctions [34]. Along with increased vascular permeability, histamine also causes edema formation and improves the gastric acid secretion [28]. Histamine at larger concentration causes swelling of endothelial cells and leukocyte adherence. Thus, histamine is main mediator that causes early vascular changes during inflammatory response [35]. Serotonin another vasoactive amine is mainly found in the tissues of intestine, brain, and platelets which causes an increased vascular permeability and the contraction of smooth muscles [34]. Serotonin elicits venous constriction and at higher concentration slows the capillary flow and leads to stasis [35]. Bradykinin leads to endothelial cell separation, the formation of gaps in post-capillary venules and augmented vascular permeability [34].

### 4.4. Hydrolytic Enzymes

During the course of inflammation, the secretion of stored proteolytic enzymes occur from stimulated proinflammatory cells. Elastin is a primary elastic component of blood vessels, lungs, and proteins including collagen, proteoglycans, and immunoglobulins. Release of human leukocyte elastase (HLE) from the stimulated polymorphonuclear leukocyte (PMNL) leads to elastin hydrolysis and endothelial migration of stimulated proinflammatory mediators [28].

### 4.5. Reactive Oxygen Species (ROS)

Inflammation and oxidative stress are interrelated to pathophysiological events in numerous diseases [36]. Reactive oxygen species (ROS) has the important role in cellular defense mechanisms. ROS released from inflammatory cells exaggerate the oxidative stress [36,37]. ROS can initiate intracellular signaling pathways and promote proinflammatory gene expression [36,38,39]. Overproduction of radicals and peroxides impairsthe endogenous anti-oxidative mechanisms and free radical scavengers which causes deterioration of functionally relevant structures [28].

### 4.6. Transcription Factors

Nuclear factor-kappa beta (NF-κB) is the chief regulator of both the immune system and the inflammatory response [40]. NF-κB controls the transcription of genes involved in apoptosis, cell adhesion, proliferation, cellular stress response, immune response, inflammatory pathways and tissue remodeling [40]. NF-κB regulates the transcription of inflammatory cytokines like IL-1β, IL-2, IL-6, IL-8, and TNF-α along with the genes encoding COX-2, iNOS, cell adhesion molecules, immune-receptors and growth factor receptors. The glucocorticoids, aspirin at high doses, and sulfasalazine decrease the activation of NF-κB. Thus, NF-κB is an interesting therapeutic target for the pharmacotherapy of inflammatory ailments [41].

### 4.7. Complement System

The complement cascade activation results in the formation of anaphylatoxins C3a, C5a, and membrane attack complex [2]. C5a is a potent chemoattractant that causes enhanced antibody production; synthesis, and release of cytokines, PGs and leukotrienes and oxidative stress. It also favors the recruitment of inflammatory cells like neutrophils, eosinophils, monocytes and T lymphocytes [42]. Thus, complement-activated products like C5a exhibit dominant biological activities which initiate the inflammatory cascade. Several mediators of inflammation are summarized in Table 1.

## 5. Inflammation as a Therapeutic Target of Phytoconstituents

**Figure 2:**
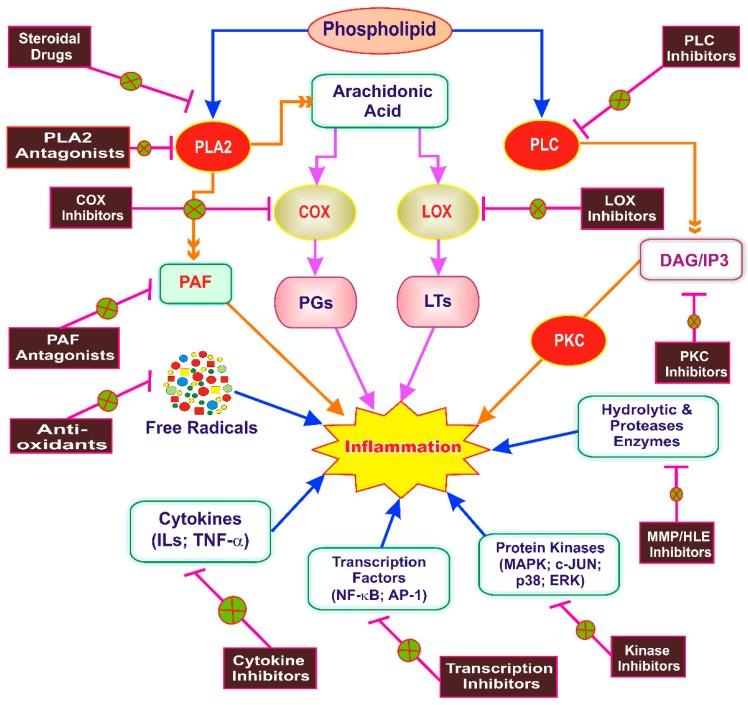
Targets of phytoconstituents in inflammation. PLA2, Phospholipase A2; PLC, Phospholipase C; PKC, Protein kinase C; PAF, Platelet-activating factor; DAG, Diacylglycerol; IP3, Inositol triphosphate; COX, Cyclooxygenase; LOX, Lipoxygenase; PGs, Prostaglandins; LTs, Leukotrienes; MMP, Matrix metalloproteinase; HLE, Human leukocyte elastase; ILs, Interleukins, TNF-α, Tumor necrosis factor-alpha; NF-κB, Nuclear factor kappa beta; AP-1, Activator protein-1; MAPK, Mitogen-activated protein kinase; P38, P38 kinase; c-JUN, c-Jun N-terminal kinase; ERK, Extracellular signal-regulated kinase. The arrows in the figure represents the process and fork represents inhibition of target.

Inflammation itself is a source of discomfort and major cause of the pathophysiological processes involved in the initiation and progression of the many diseases [59]. Systematic investigations of phytoconstituents for its anti-inflammatory activity can provide the safer and efficacious remedies to treat the inflammatory diseases [3]. Over the centuries, the treatment of inflammatory disorders are achieved through the use of medicinal plants. The phytoconstituents present in these medicinal plants are recognized to be responsible for their anti-inflammatory activities. The capability of phytoconstituents to act on several steps of pathophysiological processes is responsible for their anti-inflammatory activity. The phytoconstituents exhibit potential anti-inflammatory activities by interaction with important cellular targets including the inflammatory pathways or specifically with certain components of the pathways like the proinflammatory mediator production, complement cascade activation, and leukocyte migration [59]. Different biomolecules like matrix-degrading enzymes, proinflammatory cytokines, and the components of signaling pathways are the promising therapeutic targets in chronic inflammatory diseases [2]. Regulation of pro-inflammatory substance gene expression is the key target of phytoconstituents during an inflammatory process [3]. Safayhi et al. [28] proposed that anti-inflammatory compounds might act by one or more of the several mechanisms. Blockade of proinflammatory mediator biosynthesis, reduced expression of key enzymes, inhibition of mediator release, blockade of the interaction between mediator and its receptors are few to summarize. Various targets of phytoconstituents in inflammation are depicted as Figure 2.

## 6. Anti-Inflammatory Drug Discovery from Phytoconstituents: Current Status and Systemic Approach

Majority of drugs existing in the market constitute the natural products and even the semi-synthetic or synthetic drugs have originated from the natural sources. The therapeutic potential of medicinal plants used in several traditional systems has been established through scientific studies from across the globe. The interest of the scientific community in correlating the phytoconstituents of a plant and their botanical properties with its pharmacological activity has increased [60]. The plant extracts and isolated phytoconstituents have expressively contributed to the new drug discoveries. Finding viable, robust, and druggable lead candidates is a challenging task in drug discovery and development of pharmaceuticals for use in humans. As it involves the transformation of screening hits to the drug candidate, it demands for both experience and expertise [61]. The new drug development is a very expensive, time-consuming, and complex task. Usually, it takes about 12 years from the discovery of new lead to its appearance in the clinic as a therapeutic agent. The diminution in the new drug approvals and escalating development cost are major challenges in the new drug discovery. Although the arrival of combinatorial chemistry has rationalized the drug discovery process, it does not increase the success rate. Primarily, drug discovery focus on the identification of new chemical entities possessing potential characteristics of druggability [62]. Natural products have formed the basis of useful therapeutic agents for centuries. Plants have continued to serve mankind with discoveries of new remedies [15]. Several phytoconstituents like flavonoids, triterpenoids, alkaloids, steroids, and phenols have been documented to possess interesting anti-inflammatory properties. Many phytoconstituents exhibited potent activities at micromolar concentrations against well-established biomarkers of inflammation (Table 2). The active components obtained from natural products used as traditional medicines appear to be the main sources of drug discovery in modern medicines. Despite the advances in the allopathy field, plants are still continue to be the source of potential therapeutic agents in the modern and traditional system of medicine. Therefore, the isolation of pure compounds from the natural sources, and characterization of pharmacologically active compounds have been continued [15]. The chemical diversity and advancements in new technologies have transformed the drug discovery from natural sources. Traditional limitations of the natural products have been overcome by the novel technologies. It has created an avenue to establish the value of natural products as a drug discovery leads [61]. Structural interpretation of phytoconstituents has enabled the medicinal chemist to synthesize the compounds by total synthesis instead of their isolation from the plants. This has been resulted in the decreased production cost and improved potency of natural leads [63].

The natural product or phytoconstituent-based drug discovery has successfully introduced the important drug molecules like artemisinin, silymarin, vincristine, and vinblastine to treat malaria, hepatic diseases, and cancer, respectively. The World Health Organization has also suggested the development of plant-based medicines in conditions lacking safe synthetic drugs [15,100,101,102]. Clinical trials are ongoing on many natural products and several compounds are still in the discovery pipeline. The severalcompounds at the development phase are sourced from the plants and microorganisms. Although phytoconstituent drug development and discovery is predominantly directed towards the search of drugs effective in cancer, infectious diseases, cardiovascular diseases, gastrointestinal diseases, inflammatory diseases, and metabolic diseases [62].

In the milieu of anti-inflammatory drug discovery, interest has been directed to the phytoconstituents that have a reputation as remedies in the traditional medicine for inflammatory disorders [27]. Recently, the search for anti-inflammatory phytoconstituents has been increased. Herbal extracts possess a diversity of components or secondary metabolites having multiple biological activities [4]. Limitations of drug discovery and development from natural sources include the complex nature of extracts, isolation of phytoconstituents with considerable purity, and the lowest yield of active phytoconstituents from plants. Despite the hurdles in phytoconstituent drug discovery, plants are interesting targets for the hunt for novel anti-inflammatory leads. Identification of new compounds with desirable activity and pharmacokinetic properties is the core challenges in the new drug development. The phytoconstituents have certain unique features like more chiral centers, more oxygen atoms, molecular rigidity, steric complexity, and more hydrogen bond donors and acceptors [62,103]. As medicinal chemists are working towards the development of analogs, these unique features of phytoconstituents pose a problem in structural activity studies. It is assumed that despite being biologically active and having favorable pharmacokinetic properties, the phytoconstituents fail to satisfy drug-likeness criteria. Lipinski’s rule of five (ROF) forms the basis for the drug candidates reaching phases of the clinical trials. Although the breakthrough molecule paclitaxel does not pass the ROF, it has been clinically successful. This necessitates the design of alternative druggability criteria for the phytoconstituents [62].

**Figure 3:**
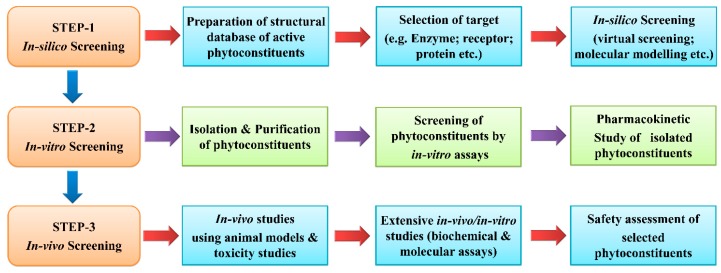
A systematic approach for preclinical evaluation of phytoconstituents.

Butterweck et al. [104] suggested that preclinical screening of natural products should begin with the in-vivo evaluation of crude extracts in the appropriate animal models so as to validate its traditional use, followed by bioassay-guided fractionation processes through the use of in-vitro model. Subsequently, the pharmacokinetics and in-vivo studies of isolated compounds should be executed [104]. The chances of new lead discovery from the natural sources should be significantly better than the chances for the new lead discovery from synthetic high-throughput drug discovery program [104]. Despite the accessibility of enormous data on anti-inflammatory plants, we are still counting for the newer ones. Many phytoconstituents have been studied extensively and reached up to clinical trials. The current drug discovery scenario suggests the re-evaluation of promising plant products (preferably isolated phytoconstituents) with the help of extensive biochemical and molecular techniques to explore the therapeutic potential and safer anti-inflammatory leads. In the framework of anti-inflammatory drug development, it is logical to investigate the possible biochemical mechanisms underlying the activities of phytoconstituents to inculcate their mechanisms of action [27]. The systematic approach for anti-inflammatory drug development from phytoconstituents is proposed as Figure 3.

## 7. Anti-Inflammatory Drug Development from Natural Products: Current Status and Systematic Approach in Use of Different Animal Models for Evaluations

The role of pharmacology in modern medicine is to search new therapeutic drugs by using appropriate models and elucidate the mechanism for therapeutic targeting by other novel molecules. The experimental models based on pharmacological principles should provide physiologically and clinically relevant model system to predict the intended therapeutic indication. A pharmacological model can be considered relevant when the effects obtained in the preclinical model are linked with the results in the clinical setting. Vogel et al. [105], described various in-vivo and in-vitro methods for the pre-clinical assessment of anti-inflammatory drugs. Before the execution of actual assay, it should be planned appropriately concerning the sample size, statistical methods, route of administration, and use of positive control [104]. Although many phytoconstituents are studied for anti-inflammatory activity, studies involving delineation of mechanisms of action, pharmacokinetics, and safety of phytoconstituents are still desirable. Inappropriate planning and execution of the drug screening program seem to be the main reason behind the declined success of phytoconstituents based drug discovery programs. While selecting animal models for the screening of anti-inflammatory activity, consideration should be given to correlate mechanisms behind well-established animal models.

### 7.1. Animal Models and Mechanisms for Screening of Anti-Inflammatory Activity

#### 7.1.1. Acute Inflammation

***•  Carrageenan-Induced Paw Edema***

Carrageenan-induced paw edema model is widely used to assess the anti-inflammatory activity of several natural and synthetic compounds [106,107]. It is the distinctive model of the acute inflammation having greater reproducibility [108,109]. Carrageenan is a non-antigenic phlogistic agent with the devoid of any visible systemic effects [109]. Sulphated sugars present in carrageenan are liable for the activation of complement system and the inflammatory mediators [110]. Stimulation of phospholipase A_2_ by carrageenan initiates the early phase of inflammation, whereas the cytotoxic effects progress the inflammation [111]. Carrageenan dilates postcapillary venules that result in exudation of inflammatory fluid and cells. This process involves the release of several proinflammatory mediators. These events represent the early exudative inflammatory phase and its inhibition terminate the inflammatory process [112]. Carrageenan model is typically linked with the activation of the cyclooxygenase pathway. Glucocorticoids and prostaglandin antagonist exhibit anti-inflammatory activity in this preclinical model [113]. The edema developed by carrageenan is represented as biphasic curve [114]. The first phase of carrageenan-induced inflammation is partly assigned to the injection trauma and released of acute phase mediators especially the serotonin and histamine [115]. Prostaglandins are the main players for the occurrence of second phase of carrageenan-induced inflammation, which occurs around 3 hr after carrageenan injection [116].

***•  Histamine/5-HT-Induced Paw Edema***

Histamine and 5-HT-induced paw inflammation models are employed for the screening of various anti-inflammatory compounds. Histamine is significant mediator of acute inflammation. Histamine and 5-HT promotes the vascular permeability and act with prostaglandins to induce inflammation [117,118,119]. Subplantar administration of histamine causes the outflow of fluid and the plasma proteins into the extracellular spaces. It increases the flow of lymph and the subsequent development of edema. Histamine acts on the H_1_ receptors and causes the contraction and separation of endothelial cells at their boundaries which increases the vascular permeability [37,119,120]. Histamine also releases neuropeptides and prostaglandins, leading to hyperalgesia and inflammation [22]. Acute phase mediators like 5-HT increase the vascular permeability by producing inter-endothelial gaps [121]. These mediators are present in the mast cell granules and are released upon its stimulation. These mediators act through the receptors present on adjacent vasculature and causes plasma extravasation [122].

***•  Bradykinin-Induced Paw Edema***

The bradykinin-induced paw edema is partly mediated by the prostaglandins (PGs). It is proposed that stimulation of phospholipase activity by the bradykinin promotes the biosynthesis of PGs. The capability of indomethacin to inhibit bradykinin-induced paw edema justifies the involvement of PGs in the formation of bradykinin induced paw edema. Incubation of human endothelial cell cultures with bradykinin releases arachidonic acid metabolites [123,124].

***•  Dextran-Induced Paw Edema***

Dextran-induced paw edema model involve increased vascular permeability, kinins activation, the release of mediators like histamine and serotonin, which develop the osmotic edema having minimal neutrophils and proteins [59,125,126]. Dextran administration leads to rapid and transient development of edema [123,127]. The liberation of histamine and serotonin following dextran administration results in the interaction of these mediators with respective receptors (H1, H2, and 5HT2) [23,123,127].

***•  Lipopolysaccharide (LPS)-Induced Paw Edema***

LPS is known to induce time-dependent rise in the TNF-α, IL-1β expression and myeloperoxidase activity in the mouse paw [128]. LPS-induced paw edema assist in the identification of drugs that are effective against TNF-α mediated inflammation. Subplantar injection of LPS into the rat paw causes an acute localized inflammatory reaction and swelling of injected paw [128,129].

***•  Arachidonic Acid-Induced Ear Edema***

Animal models of cutaneous inflammation are employed to identify the compounds that are valuable for the treatment of inflammatory diseases of the skin [130]. The ear inflammation model is valuable for assessing the anti-inflammatory potential of synthetic compounds and herbal extracts [131]. Arachidonic acid-induced mouse ear edema is facilitated by arachidonic acid metabolites like prostaglandin E2 (PGE2) and leukotriene C4 (LTC4) [132]. Earlier studies demonstrated that the arachidonic acid application by topical route leads to its rapid conversion into cyclooxygenase and lipoxygenase products. Thus, the edema reduction by any pharmacological agents is correlated with the reduction in lipoxygenase or cyclooxygenase product formation [133]. Topical application of AA produces eicosanoids like leukotrienes, prostaglandins, and thromboxanes that causes visible symptoms of inflammation [130]. Inflammation is manifested as intense erythema, edema, and accumulation of neutrophils. Moreover, local treatment with AA causes increased IL-1β expression [134]. Formed eicosanoids are also accountable for the degranulation of mast cell and subsequent release of histamine. Therefore, the anti-inflammatory activity demonstrated by compounds in this model is correlated with the antihistaminic and antioxidant property of the compounds [130].

***•  Croton oil/TPA-Induced Ear Edema***

Ear edema caused by croton oil or its irritant principle 12-O-tetradecanoylphorbol-13-acetate (TPA) has been extensively used to assess the anti-inflammatory activity of steroidal and non-steroidal anti-inflammatory drugs [132]. TPA-induced ear inflammation represents the model of skin inflammation useful for the appraisal of systemic and local anti-inflammatory compounds [135]. Topical administration of croton oil causes vasodilatation, promote vascular permeability, neutrophil influx, synthesis of eicosanoids, and liberation of serotonin and histamine [130]. TPA model is valuable for the screening of herbal extracts and synthetic anti-inflammatory compounds [136]. PGI2 and LTB4 are mainly accountable for augmented vascular permeability. Thus, the COX and LOX inhibitory compounds have been expected to inhibit the TPA-induced inflammation [136]. Furthermore, the proinflammatory effects of TPA are facilitated by the stimulation of protein kinase C that subsequently activates other enzymes like mitogen-activated protein kinases (MAPKs) and phospholipase A2. The COX, LOX, and PLA2 inhibitors and corticoids have the capability to suppress the inflammation which occurs after topical application of TPA [16,130].

***•  Oxazolone-Induced Ear Edema***

The oxazolone is most frequently used allergen for the initiation of delayed-type hypersensitivity (DTH). Oxazolone increases CD8+ T-lymphocytes and produces skin sensitization. Topical application of oxazolone leads to elevated arachidonate metabolites like prostaglandins and leukotrienes in the tissue. It also enhance the NOS-2 expression in keratinocytes. The corticosteroids and specific cytokine expression inhibitors have ability to inhibit the oxazolone-induced DTH and control eicosanoids levels [137,138]. Repeated oxazolone application to the ear of experimental animal induces chronic contact dermatitis. It is represented by continued ear swelling, noticeable inflammatory cell infiltration and prominent epidermal hyperplasia. Additionally, oxazolone causes a marked increase in interferon-γ (IFN-γ) level and minimal alterations in the IL-4 level. Interferon-γ has the capability to cause the activation of various inflammatory cells. It increases keratinocyte proliferation and causes epidermal thickening [139,140].

***•  Acetic Acid/Compound 48/80-Induced Vascular Permeability***

This test is used to study the inhibitory activity of drugs against augmented vascular permeability induced by phlogistic agents like acetic acid and compound 48/80. The compound 48/80 is mast cell degranulator and a potent activator of histamine release [141,142]. During inflammation, vascular permeability rises to permit the plasma constituents like antibodies and complement to access the infected or injured tissues. Mast cell stimulation causes the release of mediators, including histamine, prostaglandins, and leukotrienes. It increases vascular permeability through the dilation of arterioles and venules [143]. The plasma proteins and fluids are extravasated due to raised vascular permeability and subsequent edema. The increased vascular permeability is assessed through the infiltration of Evan’s blue dye at the injected sites [144].

***•  Pleurisy Model***

Pleurisy represents an exudative inflammation [145]. Pleurisy is induced in the laboratory animals by various phlogistic agents like carrageenan, dextran, compound 48/80, enzymes, and antigens. Carrageenan-induced pleurisy is established experimental model of acute inflammation. In this model, it is possible to evaluate the inflammatory phenomenon like fluid extravasation, the leukocyte migration and biochemical parameters in the exudate [144,145]. In this model, the quantification of pleural exudates, the total protein content and inhibition of leukocyte migration demonstrates the acute anti-inflammatory effect of test compounds [146].

#### 7.1.2. Sub-Acute Inflammation

***•  Granuloma Pouch Model***

Administration of the irritant substances into the subcutaneous air pouch produces proliferation of granulation tissue. The tissues mainly comprises of an endothelial cells and fibroblasts. Additionally, administration of irritant substance causes macrophage and polymorphonuclear leukocyte infiltration. In this model, there is possibility of exposure of growing tissue to the carcinogenic and the mutagenic substances. It is advantageous to administer the test substances into an air pouch because it causes direct contact of test compounds with the target cells [144].

#### 7.1.3. Chronic Inflammation

***•  Cotton Pellet-Induced Granuloma***

This model represents the pathological events in chronic inflammation. It is a widely used model for the assessment of chronic anti-inflammatory activity of newer compounds [127]. It is represented by monocyte infiltration, fibroblast proliferation, angiogenesis, and exudation [147]. The transudative and proliferative elements of the chronic inflammation are evaluated through this model. Granulomatous tissue formation is the hallmark of a chronic inflammatory process [148]. The proliferation of macrophages, neutrophils, and fibroblasts along with the multiplication of small blood vessels produces highly vascularized reddish mass, referred as granulation tissue [149]. The fluid absorbed by the pellet greatly affect the moist (wet) weight of granuloma. The moist and dry weight of cotton pellet is linked with the transudate and granulomatous tissue formation respectively [150]. Corticosteroids inhibit the inflammation at proliferative phase [151].

***•  Formalin-Induced Paw Edema***

This model has close resemblance with human arthritis. It is considered as the suitable experimental model for the assessment of chronic anti-inflammatory effect of various agents [152]. Formalin induced inflammation is biphasic. The early neurogenic phase is conciliated by bradykinin, and substance-p whereas the later inflammatory phase shows the involvement of histamine, 5-HT, prostaglandins, and bradykinin [142]. The CNS acting drugs like opioids suppress both the phases equally. While the drugs acting through the peripheral nervous system like NSAIDs and corticosteroids exclusively inhibit second phase [153].

***•  Complete Freund’s Adjuvant (CFA)-Induced arthritis***

The CFA-induced arthritis model in experimental animals signify chronic inflammation involving several systemic changes along with synovial hyperplasia [154]. It results from massive leukocytes infiltration, increase in chemokine and cytokine levels including IL-1β and TNF-α, release of ROS, cartilage and bone destruction, along with swelling and deformation [154,155,156]. CFA injection into the footpad of rat causes swelling of periarticular tissues like ligaments and joint capsules. Edema induced by CFA increases gradually at an early phase of the inflammation which becomes constant within two weeks. The decrease in CFA-induced paw inflammation is an index of anti-inflammatory effect of test drug. Measurement of injected (ipsilateral) and non-injected (contralateral) paw edema supplemented with antioxidant estimations, visual arthritis scoring system, nitrite content determination, hematological and biochemical evaluations along with radiological and histopathological studies assist to establish the probable mechanisms behind the anti-inflammatory and analgesic activities of tested compounds [157,158,159].

**Figure 4:**
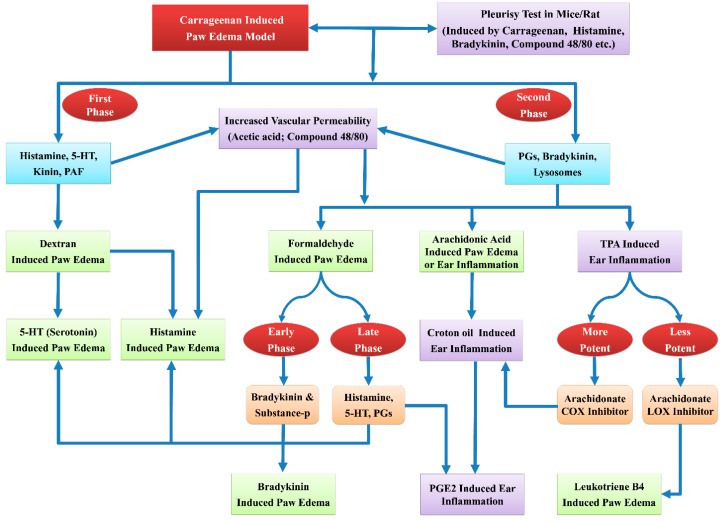
Scheme for the preclinical evaluation of the acute anti-inflammatory activity.

The scheme presenting some widely used acute models of anti-inflammatory activity is given as Figure 4.

### 7.2. Advantages and Limitations of Animal Models of Inflammation

#### 7.2.1. Carrageenan-Induced Paw Edema

- **Advantages:** Widely used and well-established working model of the inflammation [109]. Inflammation-induced by carrageenan is acute, non-immune, and reproducible [109]. Involvement of multiple mechanisms allow this model as a preliminary test for the screening of anti-inflammatory drugs [160]. Biphasic response after subplantar carrageenan injection enable this model to predict the probable biological targets of test drug in the inflammation. This model is sensitive to cyclooxygenase inhibitors and suitable for the assessment of NSAIDs that act by the cyclooxygenase inhibition which is involved in prostaglandin synthesis [109,161].
- **Limitations:** To eradicate the effects of stress, animals should be acclimatized at least one week before the commencement of an experiment [162]. The investigator should be trained to record the stable and reproducible paw volumes using sophisticated equipment like plethysmometer. Rise in paw edema is based on the concentration of injected carrageenan. Typically the maximum edema response produced by carrageenan is too difficult to inhibit. Therefore, the carrageenan type and preparation of its solution needs careful attention [160,162].

#### 7.2.2. Histamine/5-HT-induced Paw Edema

- **Advantages:** Histamine/5-HT-induced paw edema methods are convenient to appraise the acute anti-inflammatory effect of substances. These models can be used as secondary models to authorize the results of carrageenan-induced paw edema model, especially for those drugs that showed effect at the first phase of carrageenan-induced inflammation. These models are suitable for the assessment of those drugs that act through the histamine and/or 5-HT inhibition [163,164].
- **Limitations:** Inflammation or paw edema induced by injection of histamine or 5-HT is minimal and transient. These models are rather inappropriate for the assessment of drugs like prostaglandin inhibitors, which act by the mechanisms excluding histamine and/or 5-HT [163].

#### 7.2.3. Bradykinin-induced Paw Edema

- **Advantages:** It is an animal model of the acute inflammation. Results of this model can be correlated with the results of carrageenan-induced paw edema model. Drugs inhibiting prostaglandins are effective in this model.
- **Limitations:** Bradykinin produces only mild and transient edema [123,124].

#### 7.2.4. Dextran-induced Edema

- **Advantages:** This model includes both histamine and the serotonin for the induction of edema. Therefore, it is suitable to assess the anti-inflammatory effect of anti-histaminic or anti-serotonin drugs. This model is employed to reinforce the results of carrageenan-induced paw inflammation model.
- **Limitations:** This model is unsuitable for drugs that act through the mechanisms other than anti-histaminic or anti-serotonin [59,125,126].

#### 7.2.5. Lipopolysaccharide-Induced Paw Edema

- **Advantages:** This model is very suitable for the recognition of anti-inflammatory agents that acts through cytokine modulation [129,165]. Besides, paw inflammation, lipopolysaccharide also induces inflammatory hyperalgesia. Therefore, this model is advantageous for the simultaneous assessment of analgesic and anti-inflammatory drugs [128].

#### 7.2.6. Arachidonic Acid-Induced Ear Edema

- **Advantages:** Valid model of topical acute inflammation. This is beneficial for the recognition of anti-inflammatory compounds that acts through eicosanoids inhibition.
- **Limitations:** Generally, animals are sacrificed at the end of experiment [131,133].

#### 7.2.7. Croton Oil/TPA-Induced Ear Edema

- **Advantages:** These models are appropriate for the screening of steroidal and non-steroidal anti-inflammatory drugs. In the TPA induced ear edema model more potent and less potent results are linked with COX and LOX inhibitors, respectively.
- **Limitations:** As multiple mechanisms are involved in the croton oil/TPA induced ear edema model, it is rational to use these models to only predict rather than to approve the mode of action of anti-inflammatory compounds. Usually, animals are sacrificed at the completion of the experimental protocol to harvest the tissue samples and the auricular lymph nodes for detailed investigations [130,132].

#### 7.2.8. Oxazolone-Induced Ear Edema

- **Advantages:** Well recognized model of delayed-type hypersensitivity (immune inflammation).
- **Limitations:** Possibility of exclusion of anti-inflammatory compounds that act exclusively through non-immune mechanisms from early drug development programs. Frequently, animals are sacrificed at the end of the experiment [137,138,139,140].

#### 7.2.9. Acetic Acid/ Compound 48/80-Induced Vascular Permeability

- **Advantages:** Appropriate experimental model for the assessment of acute anti-inflammatory effect. The ant-inflammatory activity of drugs against compound 48/80-induced vascular permeability is linked with mast cell stabilization or anti-histaminic activity of drugs.
- **Limitations:** Intraperitoneal injection of acetic acid causes severe irritation which is inappropriate with respect to animal welfare and may raise ethical issues. Frequently, animals are sacrificed at the completion of the experiment [143,144].

#### 7.2.10. Pleurisy Tests

- **Advantages:** These tests are suitable to screen the acute anti-inflammatory activity. This test enables to evaluate the inflammatory phenomenon like fluid extravasation, the leukocyte migration and biochemical parameters in the exudate.
- **Limitations:** These methods inflict severe pain and may be accompanying with systemic infections if sterility is not observed during injection of the phlogistic agents. Usually, animals are sacrificed at the termination of experiments [144,145].

#### 7.2.11. Granuloma Pouch Model

- **Advantages:** Model of sub-acute inflammation. It is beneficial to administer the test substances into the air pouch because it causes direct contact of test compounds with the target cells.
- **Limitations:** Subcutaneous injection of a large volume of air induces pain to experimental animals. The procedure requires anesthesia. Usually, animals are sacrificed at the end of the experiment [144].

#### 7.2.12. Cotton Pellet-Induced Granuloma

- **Advantages:** Widely used model of the chronic inflammation. Wet/moist weight of granuloma correlates with the amount of transudate. The dry weight of granuloma relates with the granulomatous tissue formation. Generally, steroidal drugs inhibit dry weight of granuloma. The biochemical analysis of granuloma provides information about the proliferative changes occurring due to chronic irritation and inflammation. Hence, this model provides a window to additional markers of the inflammatory process.
- **Limitations:** Implantation and removal of cotton pellet and granuloma needs anaesthetics and surgical skills. The implantations may cause localized sepsis and this may confound the observations. Repeated handling of the animals, removal of surgical stitches, and need of sacrifice of the animals to collect the granuloma are other shortcomings of this model [150,151].

#### 7.2.13. Formalin-Induced Paw Edema

- **Advantages:** This is the experimental model of chronic inflammation that closely resemble human arthritis. Depending on the capacity of the drug to act on the neurogenic phase, the inflammatory phase or on both the phases it is possible to predict the involvement of central or peripheral components in the anti-inflammatory effect of drugs.
- **Limitations:** Formalin is a severely irritating agent and exposes the experimental animals to severe pain [142,153].

#### 7.2.14. CFA-Induced Arthritis

- **Advantages:** Well-characterized model of the chronic inflammation and arthritic alterations. Though seronegative in nature, CFA-induced inflammation involves immune-inflammatory components. Inflammation in the CFA-injected (primary lesions) and non-injected (secondary lesions) paws represent the clinical symptoms of human inflammation and arthritis, respectively. This model can be used to assess the effect of test drugs against acute, chronic and immune-inflammatory and arthritic conditions.
- **Limitations:** Preparation and induction of CFA may affect the severity of arthritic response and therefore need careful attention. The evaluations need sophisticated instruments like plethysmometer and Von-Frey apparatus to measure alterations in the paw volume and pain threshold. As the experimental duration is more and it involves painful and inflammatory pathology, it is stressful to the animals. Generally, animals are sacrificed at the completion of the protocol [154,157].

### 7.3. In-Vitro Detection of Inflammatory Biomarkers

In-vitro bioassays are the important tools in the pharmacological evaluations of natural products especially the phytoconstituents. The applications of in-vitro methods for the screening of botanicals include bioassay guided fractionation, pharmacological and biological characterization, interaction studies, assistance in stability studies and the identification of mode of actions [166]. Overall, the choice of any evaluation tool either in-vitro bioassays or in-vivo models are governed by the background data available on the phytoconstituents to be evaluated and the goal of the research program [166].

Lipopolysaccharide (LPS) stimulated RAW 264.7 macrophages and human monocytic leukemia cells (THP-1) were usually used to assess the anti-inflammatory effect of phytoconstituents. The stimulation of such cells with LPS produces proinflammatory cytokines (IL-1, IL-6, TNF-α) and chemokines (IL-8, MCP-1). The cells are either directly pre-treated with the supernatant of extracts (after through centrifugation) or with pure photochemical followed by LPS stimulation [104]. The severity of inflammation is assessed through the manifestation of pro-inflammatory mediators like TNF-α, IL-1β, nitric oxide (NO) and cyclooxygenase (Cox-1 and Cox-2) in cell supernatants [22]. The enzyme immunoassay (EIA) kits can be used to quantify the mediators like PGE2, interleukins, prostaglandins, and TNF-α [22]. The cytokine levels can be measured in-vitro using sandwich ELISA kits [167]. Expression of pro-inflammatory mediators is controlled by several signaling pathways and transcription factors. Among these pathways, mitogen-activated protein kinases (MAPKs) regulate the cell growth, differentiation, apoptosis and cellular response to cytokines. The MAPK pathways include p38 MAPK, extracellular signal-regulated kinase (ERK) and c-Jun NH2-terminal kinase (JNK). The MAPK inhibitors suppress the expression of iNOS gene. The iNOS expression could also be modified by phosphatidylinositol-3-kinase (PI3K)/Akt pathway stimulated by cytokines and certain growth factors. The MAPKs and Akt also play a critical role in the activation of another important transcription factor termed as NF-κB [104].

The western blotting assay is useful to measure the expression of transcription factor proteins like p38 MAPK, JNKs, ERK, Akt, IKKα/β, and IkB-α. It includes extraction of cellular proteins from the cells pre-treated with test substances, cell lysis, electro-blotting and separation on a sodium dodecyl sulfate (SDS)-polyacrylamide gel electrophoresis. The immunoblots are treated with primary antibodies and suitable secondary antibodies (usually horseradish peroxidase-conjugated antibodies) with successive washings. The blots are developed by chemiluminescence technique to assess the protein expressions [22,86].

### 7.4. Biomarkers for Prediction of Side Effects of Anti-Inflammatory Drugs

Anti-inflammatory drugs like NSAIDs are usually associated with various side effects including gastrointestinal and cardiovascular adversities. Prediction of an adverse effect profile of novel anti-inflammatory drugs during the early development stage could be beneficial to understand the type and severity of adverse effects possessed by the drug under development. Tools like biomarkers estimation and metabolomics assists to predict the adversity of anti-inflammatory drugs during their developmental stage. Identification, quantification, and prediction of drug toxicity have been possible through the use of various biomarkers of toxicity [168].

#### 7.4.1. Biomarkers for Prediction of Gastrointestinal Side Effects

Currently, three main biomarkers, namely blood, fecal, and orally administered probes, are available for the prediction of gastrointestinal toxicity of drugs. The various biomarkers used to predict the gastrointestinal side effects of drugs are discussed.

***•  Citrulline***

Citrulline is non-protein amino acid produced by enterocytes of small intestine and precursor for the renal arginine synthesis [169]. Citrulline is detected in the plasma or serum as a circulating free amino acid. Citrulline is suggested safety biomarker of an enterocyte mass and function used to assess small intestinal toxicity in laboratory animals and humans [170,171,172]. Although analysis of citrulline in the blood is limited by less sensitive enzymatic assays, application of techniques like high-performance liquid chromatography (HPLC) and mass spectrometry (MS) enables the circulating citrulline as a detectable biomarker of gastrointestinal safety [173,174].

***•  Diamine oxidase (DAO)***

Diamine oxidase is a degradative enzyme which catalyzes oxidation. It is located in the intestinal mucosa at the upper villus cells [173,174,175]. Maturity and integrity of small intestinal mucosa are represented by serum DAO activity. DAO is the predictor of gastrointestinal mucosal damage [176]. DAO activity in the small intestinal mucosa is correlated with the DAO activity in the blood [174,177]. DAO is mainly present in various tissues including small intestinal mucosa in both humans and rodents and acts as a regulator for rapidly proliferating tissues like intestinal mucosa. Therefore, DAO can be a reliable biomarker for the quantitative prediction of mucosal integrity and intestinal mucosal mass [175,178,179]. However, minute level of DAO in the blood is a limiting factor for the effective measurement of DAO. Therefore, sensitive methods are demanded the effective integration of this important safety biomarker at the pre-clinical drug development stages [174].

***•  Gastrin***

Serum gastrin levels are altered concerning gastrointestinal damage caused by factors like bacterial infections, the tumors or ulcers [174]. Serum gastrin levels were increased with the use of drugs like omeprazole [180]. Therefore, gastrin levels can be the biomarker for the gastrointestinal safety of anti-inflammatory drugs.

***•  Orally Administered Probes***

Drug-induced damage to the intestinal barrier can be monitored with the urinary excretion of an orally administered probes [173,174]. Gastrointestinal damage caused by the anticancer drug was correlated with the urine sucrose and ^51^Cr-EDTA levels measured by radioimmunoassay technique [181]. Carbon-13 sucrose breath test (13C-SBT) is a non-invasive and simple technique that allows the assessment of gut function [172,182].

***•  Calprotectin***

Calprotectin is a neutrophil-derived protein that has a role in the neutrophil defense [173]. Elevated levels of fecal calprotectin are reported in conditions like treatment with NSAIDs, inflammatory bowel disease and colonic cancer. Therefore, fecal calprotectin is designated as a sensitive and stable biomarker of the gastrointestinal inflammation [183,184]. Levels of fecal calprotectin are connected with neutrophil infiltration at intestinal mucosa and also to the severity of gastrointestinal inflammation [172,174].

***•  Lactoferrin***

Lactoferrin is a major component of the polymorphonuclear neutrophil granules which is liberated by mucosal membranes. Intestinal inflammation leads to excessive infiltration of the leukocytes and the subsequent increase in the fecal lactoferrin levels [185,186]. Hence, fecal lactoferrin can be a biomarker for the prediction of an intestinal inflammation [173].

***•  Micro RNAs (miRNAs)***

Micro RNA are endogenous non-coding RNAs functioning as post-transcriptional regulators of the gene expression [187]. The miRNAs have the potential to predict and assess the gastrointestinal damage and these micro RNAs are the emerging biomarkers for several organ toxicities including cardiovascular, liver, and gastrointestinal toxicities. The miRNAs are released after cell death in the surrounding environment and remain stable in the circulation [188]. Thus, the detection of miRNAs in biofluids is used as a tool for assessing the tissue damage following the treatment with various drugs [174].

***•  Miscellaneous***

C-reactive proteins, matrix metalloproteases, pepsinogen I and II, polymorphonuclear neutrophil elastase, and bile acids are other blood and fecal biomarkers available for the assessment of gastrointestinal toxicity of NSAIDs [172,174].

#### 7.4.2. Biomarkers for Prediction of Cardiovascular Side Effects

Several biological molecules like inflammatory biomarkers, oxidative stress biomarkers, antioxidant biomarkers, cytokines, and chemokines can be used to predict the cardiovascular side effects of various drugs including anti-inflammatory therapies. These biomarkers are valuable to predict the risk of occurrence of the cardiovascular events in human patients, healthy individuals or experimental animals. However, further insights in the specificity, selectivity, stability, and validity of such biomarkers are desired to integrate these biomarkers as a preclinical or clinical tool in the early drug development programs for the drug safety prediction.

***•  Inflammatory biomarkers***

Following oxidative stress, an advanced glycation end products (AGEs) are accumulated in the vessel wall which possesses the risk of developing the cardiovascular side effects. Accumulation of AGEs is linked with the increasing evidence of an arterial stiffness and cardiovascular mortality [189]. Angiotensin II (ANG II) is chief effector in the renin-angiotensin–aldosterone system having vasoconstrictor, anti-natriuretic and pro-inflammatory properties. Elevated levels of Angiotensin II can predict the cardiovascular adversities of the anti-inflammatory drugs including chronic heart failure and unstable angina [189]. C-reactive protein (CRP) is a nonspecific inflammatory marker which has been studied extensively in cardiovascular diseases and CRP itself mediate the athero-thrombosis [190,191,192]. Myeloperoxidase (MPO) is liberated by activated leukocytes. Increased MPO levels with or without CRP are predictive of the coronary artery disease [193]. Intracellular cell-adhesion molecule-1 (ICAM-1) and vascular cell adhesion molecule-1 (VCAM-1) are expressed on the surface of endothelial cells which can be a biomarker for the prediction of coronary artery diseases [189,190].

***•  Proinflammatory Cytokines***

Proinflammatory cytokines like interleukin-1 (IL-1), interleukin (IL-6), interferon-gamma (IFN-γ) and tumor necrosis factor-α (TNF-α) predict the cardiovascular risks including coronary artery disease, myocardial infarction, chronic heart failure, peripheral arterial disease and unstable angina [189,194]. Moreover, altered levels of the anti-inflammatory cytokines including adiponectin, interleukin-4 (IL-4), interleukin-10 (IL-10), and transforming growth factor (TGF-β) signifies the risk of development of cardiovascular toxicities [189].

***•  Oxidative stress biomarkers***

Initiation and progress of the cardiovascular diseases like atherosclerosis occurs through excessive oxidative stress [195]. An imbalance between free radical formation and protective antioxidants lead to oxidative stress [196]. Arachidonic acid peroxidation causes the production of cellular non-enzymatic products called isoprostanes. These isoprostanes are known as the biomarkers for cardiovascular disease [197]. Oxidized low-density lipoprotein (Ox-LDL) has prominent role within atherosclerotic plaque formation and subsequent atherosclerosis progression [195,198]. Lipoprotein-associated phospholipase A2 (Lp-PLA2) secreted by inflammatory cells and nitrotyrosine, which are the mediators of MPO, along with Ox-LDL are related with cardiovascular disease, myocardial infarction, transient ischemic stroke and angina [189,199,200]. The success of oxidative stress biomarkers to predict the cardiovascular toxicities of compounds remains to establish. Appropriated detection methods and extensive in-vivo studies are needed to verify the significance of these oxidative stress biomarkers as predictors of cardiovascular safety [195].

***•  Antioxidant biomarkers***

Antioxidants are compounds that act as free radical scavengers. Coenzyme Q10 (CoQ10) is the antioxidant present in all the cellular membranes [189]. Decreased level of CoQ10 is linked with the development of the cardiovascular disease. Glutathione (GSH) is an intracellular antioxidant essential for the immune cells functioning [201]. Low level of GSH is correlated with chronic heart failure and myocardial infarction [189,202,203]. Superoxide dismutase (SOD) is another antioxidant enzyme which is present on the surface of the vascular lumen. Altered level of SOD is a hint for the prediction of cardiovascular side effects of drugs [189].

#### 7.4.3. Metabolomics for Prediction of Side Effects

Systematic analysis of the low molecular weight metabolites in the biological specimens, like blood, urine, saliva, tissues, and breath, is called metabolomics. Metabolomics is a vital and widely applied method for therapeutic and safety biomarker discovery [204,205]. Nuclear magnetic resonance (NMR) and mass spectrometry (MS) are widely used for metabolomic profiling [205]. However, liquid chromatography (LC), gas chromatography (GC), capillary electrophoresis (CE) hyphenated to MS, reversed-phase liquid chromatography (RPLC), mass spectrometry imaging (MSI) including secondary ion mass spectrometry (SIMS) and matrix-assisted laser desorption/ionization mass spectrometry (MALDI-MS) are also applied for metabolic analyses [204,205,206]. Surrogate biomarkers for gastrointestinal (GI) damage was developed in rat using pattern recognition analysis of the (1) H nuclear magnetic resonance (NMR) spectra of the urine sample. Hence, there is the possibility of screening the gastric damage caused by anti-inflammatory drugs at the preclinical drug development stage with the use of metabolomics [207]. Other studies have postulated the likelihood of assessing the cardiovascular toxicity of various drugs using the application of metabolomics study [204,206,208].

## 8. Conclusions

The search for a newer anti-inflammatory agent is an ongoing pursuit and natural products appears to offer a great hope for getting better anti-inflammatory compounds. Current status of drug discovery from natural sources revealed that interest in the screening of phytoconstituents for anti-inflammatory and related activities has been increased. Several plants owe their pharmacological properties mainly due to the presence of secondary metabolites like flavonoids and triterpenoids. Many phytoconstituents has demonstrated their activities towards the multiple targets of inflammation at minute concentrations with lower incidences of toxic effects. In the context of current drug discovery scenario and advanced molecular techniques, it will be better to extrapolate the anti-inflammatory studies on already reported natural products rather than counting for newer ones. Earlier reports showed that most of the anti-inflammatory studies are either conducted using crude extracts (at higher doses) or isolated phytoconstituents. However, these studies on natural products are not extended up to the establishment of their molecular mechanisms and pharmacokinetics. Systematic screening of natural products through employment of appropriate animal models, widespread involvement of biochemical and molecular estimations, development of valid pharmacokinetic and safety data, etc. should be assimilated to explore the better anti-inflammatory leads from natural origin.

## Figures and Tables

**Table 1:** Different mediators of inflammation and their pathophysiological role.

Category	Mediator	Source Organ/Cells	Mechanism	Reference
Vasoactive amines	Histamine	Basophils, gastric cells, enterochromaffin cells, histaminergic nerve cells	Vasodilatation and increased vascular permeability	[43]
	Serotonin	Intestine, blood, spleen, nervous system	Vasodilatation and increased vascular permeability (less potent than histamine)	[44]
Arachidonic acid metabolites	Prostaglandins (PGs)	Formed by the metabolism of arachidonic acid by cyclooxygenases (COX)	Enhanced vascular permeability, fever, sensory nerve stimulation, and pain amplification. PGE_2_ causes production of edema and erythema	[45]
	Leukotrienes (LTs)	Formed by the metabolism of arachidonic acid by lipoxygenase (LOX)	LTB4 stimulates neutrophil chemotaxis, enhanced neutrophil-endothelial interactions, neutrophil activation, degranulation and release of various inflammatory mediators, enzymes and free radicals	[29]
	Thromboxane (TX)	Granuloma tissues, macrophages, human synovial tissues, Thromboxane A2 (TXA2) is present in platelets and circulating leukocytes	Platelet aggregation, smooth muscle contraction	[46]
Platelet activating factor (PAF)	PAF	Liberated by macrophages, endothelial cells, platelets and neutrophils	Initiates cardinal features of inflammation, expression of adhesion molecules, platelet aggregation, formation of leukotrienes, chemotaxis, sensitization of sensory nerves to pain	[47,48]
Kinins	Substance p (Sub-p)	Released from sensory nerves	Increased microvascular permeability, neutrophil accumulation, potentiates responses to bradykinin, serotonin, prostaglandin, and ATP	[49,50]
	Bradykinin	Plasma precursor protein kininogen produces bradykinin through kallikrein	Increased vascular permeability, sensory nerve ending stimulation, inflammatory mediator release, activation of NF-κB, induction of cytokine gene expression	[50,51]
Cytokines	Interleukins (ILs)	Produced by activated lymphocytes and macrophages	Up-regulation of adhesion molecule expression, stimulation of pro-inflammatory mediator release	[52,53]
	Tumor Necrosis Factor-α and β (TNF-α, β)	Activated macrophages/monocytes, fibroblasts, mast cells, natural killer (NK) cells, T and B lymphocytes	Stimulation of PGE2, collagenase, IL-1 production, fever, induction of acute-phase reactant protein production, adhesion molecule up-regulation, cytokine induction, chemokine synthesis	[52,53]
	Transforming Growth Factor-β (TGF-β)	T cells, platelets, monocytes	Attraction of monocytes and other leukocytes to the site of injury, increased cell adhesion	[52]
	Interferons (IFN-α and β)	IFN-α is a produced by leukocytes and IFN-β is a produced by fibroblasts	Activation of macrophages and mononuclear phagocytes	[52]
Clotting system	Thrombin	Blood	Mobilization of p-selectin, the release of chemokines, adhesion molecule expression, induction of COX-2, production of PGs, PAF and nitric oxide	[54,55]
Complement system	Anaphylotoxins C3a and C5a	Complement proteins reside as inactive forms in plasma	Potentiate inflammation by binding to receptors on mast cells, basophils, phagocytic cells, and endothelial cells	[42]
Miscellaneous	Nitric oxide (NO)	Leukocytes, endothelial cells, sensory nerve cells	Vasodilation and cytotoxicity	[56,57]
	Reactive Oxygen Species (ROS)	Phagocytic leukocytes like, neutrophils, monocytes, macrophages, eosinophils	Vascular leakage, chemotaxis, endothelial damage, oxidative stress, activation of transcription factors like nuclear transcription factor-κB (NF-κB)	[58]

**Table 2:** IC_50_ values of some phytoconstituents on various targets of inflammation.

Compound	Target	Stimulus/system	IC_50_	Reference
***Alkaloid***				
Lycoricidinol	TNF-α	LPS-stimulated murine macrophages	0.002 mg/mL	[64]
Lycorine	TNF-α	LPS-stimulated murine macrophages	0.2 mg/mL	[64]
***Curcuminoids***				
Curcumin	NF-κB	NF-κB dependent transcription inhibition in reporter assays in A549 cells	21.5 μM	[65]
Curcumin	TNF-α and IL-1	LPS-induced production of TNF and IL-lβ by a human monocytic macrophage cell line Mono Mac 6	5 μM	[66]
***Diterpene***				
Cryptotanshinone	COX-2	Pulsed ultrafiltration LC–MS screening	22 μM	[67]
Nimbiol	LOX	In-vitro soybean lipoxygenase assay	106 μM	[68]
Sugiol	LOX	In-vitro soybean lipoxygenase assay	60.7 μM	[68]
***Flavonoid***				
Amoradicin	TNF-α	LPS-stimulated TNF-α release in RAW 264.7 cells	28.51 μM	[69]
Apigenin	NO	LPS-stimulated macrophages	2.8 μM	[70]
Apigenin	NO	LPS and TNF-γ-stimulated macrophages	10.4 μM	[70]
Apigenin	NO	LPS-stimulated RAW 264.7 macrophages	10.7 μM	[71]
Apigenin	NO	LPS-stimulated macrophages	19.2 μg/mL	[72]
Apigenin	Free radical	DPPH radical scavenging assay	30.3 μg/mL	[73]
Astilbin	PGE2	LPS-stimulated RAW 264.7 cells	19.6 μg/mL (43.5 μM)	[74]
Centaureidin	LOX	Soybean lipoxygenase assay	20 μM	[75]
Centaureidin	LOX	In-vitro cyclooxygenase assay	318 μM	[75]
Cirsiliol	5-LOX	5-LOX inhibition in rat basophilic leukemia cells	0.1 μM	[3]
Dioclein	PDE4	LPS-stimulated macrophages	16.8 μM	[76]
Daidzein	NO	LPS-stimulated macrophages	40.0 μM	[70]
Daidzein	NO	LPS and TNF-γ-stimulated macrophages	81.4 μM	[70]
Engeletin	PGE2	LPS-stimulated RAW 264.7 cells	14.4 μg/mL (33.2 μM)	[74]
Genistein	NO	LPS-stimulated macrophages	16.6 μM	[70]
Genistein	NO	LPS and TNF-γ-stimulated macrophages	34.5 μM	[70]
Genistein	NO	LPS-stimulated TNF-α release in RAW 264.7 cells	5 μM	[77]
Jaceosidin	COX-2	LPS-stimulated RAW 264.7 cells	2.8 μM	[78]
Kaempferol	NO	LPS activated macrophages	10.6 μM	[79]
Kaempferol	NO	LPS induced Akt phosphorylation	30.5 μM/kg	[80]
Lantadene A	Free radical	DPPH radical scavenging activity	6.5 mg/mL	[81]
Lantadene A	Hydroxy radical	Hydroxyl radical scavenging activity	42.4 mg/mL	[81]
Lantadene A	Superoxide anion	Superoxide anion radical scavenging activity	2.5 mg/mL	[81]
Lantadene A	NO	NO scavenging assay	98.0 µg/mL	[81]
Luteolin	NO	LPS-stimulated macrophages	10.4 μM	[70]
Luteolin	NO	LPS and TNF-γ-stimulated macrophages	38.6 μM	[70]
Luteolin	NO	LPS-induced NF-κB activation	35.1 μM/kg	[80]
Luteolin	NO	LPS-stimulated TNF-α release in RAW 264.7 cells	1 μM	[77]
Luteolin	NO	LPS-stimulated macrophages	10.4 μM	[79]
Quercetin	NO	LPS-stimulated RAW 264.7 cells	11.2 μg/mL (37.1 μM)	[74]
Quercetin	PGE2	LPS-stimulated RAW 264.7 cells	19.9 μg/mL (65.8 μM)	[74]
Quercetin	TNF-α	LPS-stimulated RAW 264.7 cells	1.25 μg/mL (4.14 μM)	[74]
Quercetin	TNF-α	LPS-stimulated TNF-α release in RAW 264.7 cells	1 μM	[77]
Quercetin	LTB4	Peripheral blood mononuclear cells	2 μM	[82]
Quercetin	MMP-9	Fluorescent gelatin dequenching assay and gelatin zymography	22 μM	[83]
Rutin	Free radicals	DPPH radical scavenging assay	23.7 μg/mL	[83]
Velutin	NF-κB	Secreted embryonic alkaline phosphatase reporter assay	2 μM	[84]
***Glycoside***				
Aucubin	TNF-α	Ag-stimulated TNF-α release in rat basophilic leukemia (RBL)-2H3 mast cells	0.101 μg/ml	[85]
Aucubin	IL-6	Ag stimulated IL-6 production in rat basophilic leukemia (RBL) -2H3 mast cells	0.19 μg/mL	[85]
Baicalin	COX-1	Human osteosarcoma cell line	9.8 μg/mL	[86]
Baicalin	COX-2	Human osteosarcoma cell line	7.3 μg/mL	[86]
Hederagenin	Hyaluronidase	Hyaluronidase activity assay	280.4 μM	[87]
Hederagenin	Elastase	Porcine pancreatic elastase	40.6 μM	[87]
Ligustroside	PGE_2_	Calcium ionophores stimulated mouse peritoneal macrophages	48.5 μM	[88]
Ligustroside	TXB_2_	TXB_2_ release induced by calcium ionophore in human platelets	122.6 μM	[88]
Oleuropeoside	PGE_2_	Calcium ionophores stimulated mouse peritoneal macrophages	47 μM	[88]
***Lactone***				
Artemisolide	NF-κB	LPS-stimulated RAW 264.7 cells	5.8 μM	[89]
Artemisolide	PGE2	LPS-stimulated RAW 264.7 cells	8.7 μM	[89]
Artemisolide	NO	LPS-stimulated RAW 264.7 cells	6.4 μM	[89]
Desmethoxyyangonin	TNF-α	Okadaic acid-stimulated TNF-α release from BALB/3T3 cells	17 μM	[64]
Ergolide	NO	LPS/IFN-γ-stimulated RAW 264.7 macrophages	1.95 μM	[90]
Ergolide	PGE2	LPS/IFN-γ-stimulated RAW 264.7 macrophages	3 μM	[90]
Yangonin	TNF-α	Okadaic acid-stimulated TNF-α release from BALB/3T3 cells	40 μM	[64]
***Lignan***				
(−)-pinoresinol 4-O-b-D-glucopyranoside	NO	LPS-stimulated murine microglia BV-2	34.35 μM	[91]
(−)-syringaresinol	NO	LPS-stimulated murine microglia BV-2	15.05 μM	[91]
Isoamericanol B	NO	LPS-stimulated RAW 264.7 cells	10.3 μg/mL	[92]
***Sesquiterpene acid***				
Dehydrocostic acid	LTB4	Leukotriene B4 generation by rat leukocytes	22 μM	[93]
Dehydrocostic acid	Elastase	Elastase activity assay	43 μM	[93]
Dehydrocostic acid	PLA_2_	Bee venom PLA2 activity	17 μM	[93]
***Stilbenoid***				
Piceatannol	Elastase	Procine pancreatic elastase assay	15.6 µg/mL	[91]
Resveratrol	NO	Cytokine stimulated inducible NO synthase expression and nitrite production in human primary airway epithelial cells	3.6 μM	[94]
Resveratrol	GMCS	GMCS factor release in airway epithelial cells	0.44 μM	[94]
Resveratrol	IL-8	IL-8 release in airway epithelial cells.	4.7 μM	[94]
***Tannin***				
(+)-catechin	COX-1	Human osteosarcoma cell line	2.8 μg/mL	[86]
(+)-catechin	COX-2	Human osteosarcoma cell line	10.5 μg/mL	[86]
Catechin	Elastase	Procine pancreatic elastase assay	20.2 µg/mL	[95]
***Triterpenoid***				
2,3,19-trihydroxy-24-oxo-olean-12-en-28-oic acid	NO	LPS-induced NO production in RAW 264.7 macrophages	5.4 μM	[96]
Alphitolic acid	NO	LPS+ IFN-γ activated RAW264.7 macrophages	17.6 µM	[97]
Alphitolic acid	TNF-α	LPS+ IFN-γ activated RAW264.7 macrophages	22.7 µM	[97]
Arjunic acid/Arjuntriterpenic acid	NO	LPS-induced NO production in RAW 264.7 macrophages	20.1 μM	[96]
Arjunolic acid	NO	LPS induced NO production in RAW 264.7 macrophages	13.0 μM	[96]
Betulinic acid	Elastase	Procine pancreatic elastase assay	21.6 µg/mL	[95]
Betulinic acid	NO	LPS+IFN-γ activated RAW264.7 macrophages	8.3 µM	[97]
Betulinic acid	TNF-α	LPS+IFN-γ activated RAW264.7 macrophages	23.5 µM	[97]
Cis-coumaroyl alphitolic acid	NO	LPS+IFN-γ activated RAW264.7 macrophages	3.5 µM	[97]
Cis-coumaroyl alphitolic acid	TNF-α	LPS + IFN-γ activated RAW264.7 macrophages	5.6 µM	[97]
Emmolic acid/Ceanothic acid	NO	LPS+IFN-γ activated RAW264.7 macrophages	>36 µM	[97]
Emmolic acid/Ceanothic acid	TNF-α	LPS+IFN-γ activated RAW264.7 macrophages	>36 µM	[97]
Emmolic acid acetate	NO	LPS+IFN-γ activated RAW264.7 macrophages	14.7 µM	[97]
Emmolic acid acetate	TNF-α	LPS+IFN-γ activated RAW264.7 macrophages	>36 µM	[97]
Friedelin	LOX	In-vitro soybean lipoxygenase assay	35.8 μM	[68]
Maslinic acid	PKC	Non-radioactive detection of PKC using Raji cells	11.5 µM	[98]
Oleanolic acid	PGE_2_	Calcium ionophores stimulated mouse peritoneal macrophages	23.5 μM	[88]
Oleanolic acid	Hyaluronidase	Hyaluronidase activity assay	280.4 μM	[87]
Oleanolic acid	Elastase	Porcine pancreatic elastase assay	5.1 μM	[87]
Oleanolic acid	Elastase	Procine pancreatic elastase assay	3 µg/mL	[95]
Oleanolic acid	PKC	Non-radioactive detection of PKC using Raji cells	39.29 µM	[98]
Oleanolic acid	NO	LPS-induced NO production in RAW 264.7 macrophages	7.8 μM	[96]
Paradrymoniside	NO	LPS-induced NO production in RAW 264.7 macrophages	10.1 μM	[96]
Pyracrenic acid	Elastase	Procine pancreatic elastase assay	1.5 µg/mL	[95]
Sericic acid	NO	LPS-induced NO production in RAW 264.7 macrophages	17.2 μM	[96]
Trans-coumaroyl alphitolic acid	NO	LPS+IFN-γ activated RAW264.7 macrophages	1.7 µM	[97]
Trans-coumaroyl alphitolic acid	TNF-α	LPS+IFN-γ activated RAW264.7 macrophages	10.9 µM	[97]
Ursolic acid	PGE_2_	Calcium ionophore stimulated mouse peritoneal macrophages	60.9 μM	[88]
Ursolic acid	TXB_2_	TXB_2_ release induced by calcium ionophore in human platelets	50.2 μM	[88]
Ursolic acid	LOX	In vitro soybean lipoxygenase assay	92.8 μM	[68]
Ursolic acid	PKC	Non-radioactive detection of PKC using Raji cells	27.93 µM	[98]
Daucosterol	LOX	In vitro soybean lipoxygenase assay	108.7 μM	[68]
Escin	Hyaluronidase	Hyaluronidase activity assay	149.9 μM	[87]
***Miscellaneous***				
Crotafuran E	NO	LPS+IFN-γ-stimulated N9 microglial cells	13.9 μM	[71]
Escinol	Hyaluronidase	Hyaluronidase activity assay	1.65 mM	[87]
Mansoins F	TNF-α	LPS-stimulated THP-1 cells	19.3 μM	[99]
Senkyunolide O	COX-2	Pulsed ultrafiltration LC–MS screening	5 μM	[67]

COX, Cyclooxygenase; GMCS, Granulocyte macrophage colony-stimulating; IC50, half maximal inhibitory concentration; IFN-γ, Interferon-gamma; IL, Interleukin; LOX, Lipoxygenase; LPS- Lipopolysaccharide; LTB4, Leukotriene B4; MMP-9, Matrix metalloproteinase-9; NF-κB, Nuclear factor kappa beta; NO, Nitric oxide; PDE4, Phosphodiesterase 4; PGE2, Prostaglandin E2; PKC, Protein kinase C; PLA2, Phospholipase A2;TNF-α, Tumor necrosis factor-alpha; TNF-γ, Tumor necrosis factor gamma;TXB2, thromboxane B2.

## Abbreviations

12-HETE	12-Hydroxyeicosatetraenoic acid
12-HPETE	5-hydroperoxyeicosatetraenoic acid
5-HT	5-hydroxytryptamine
AA	Arachidonic acid
Akt	Serine/threonine-specific protein kinase
AP-1	Activator protein 1
COX	Cyclooxygenase
DAG	Diacylglycerol
EETs	Epoxyeicosatrienic acids
EIA	Enzyme immunoassay
ELISA	Enzyme-linked immunosorbent assay
ERK	Extracellular signal-regulated kinase;
HLE	Human leukocyte elastase
IFN-γ	Interferon-γ
IKK	IκB kinase
IL	Interleukin
iNOS	Inducible nitric oxide synthase
IP3	Inositol triphosphate
IRAK	Interleukin-1 receptor-associated kinase
JNKs	c-Jun N-terminal kinase
LPS	Lipopolysaccharide
LPX	Lipoxygenase
LTB4	Leukotriene B4
LTs	Leukotrienes
LXs	Lipoxins
MAPKs	Mitogen-activated protein kinases
MCP-1	Monocyte chemoattractant protein-1
MMPs	Matrix metalloproteinases
MPO	Myeloperoxidase
NF-κB	Nuclear factor kappa-beta
NIK	NF-κB inducing kinase
NO	Nitric oxide
NOS-2	Nitric oxide synthase-2
NSAIDs	Nonsteroidal anti-inflammatory drugs
p38 kinase	p38 mitogen-activated protein kinases
PAF	Platelet activating factor
PGE2	Prostaglandin E2
PGs	Prostaglandins
PI3K	Phosphatidylinositol-3-kinase
PKC	Protein kinase C
PLA2	Phospholipase A2
PLC	Phospholipase C
PMNL	Polymorphonuclear leucocyte
RIP	Receptor-interacting protein
ROS	Reactive oxygen species
TBX	Thromboxane
TNF-α	Tumor necrosis factor alpha
TPA	12-O-tetradecanoylphorbol-13-acetate
